# Feeding with Care: Caregiver Perspectives on Pediatric Gastrostomy Tubes

**DOI:** 10.3390/children12070813

**Published:** 2025-06-21

**Authors:** Fareed Khdair Ahmad, Noor F. Al-Assaf, Mohammad Alzoubi, Nada Odeh, Dina Samara, Zaid Arafat Samara, Hashim M. AlHammouri, Tahani Ahmad, Salma Burayzat, Omar Alqudah, Nadia Khamees, Tarek A. Tamimi, Awni Abu Sneineh, Yaser Rayyan

**Affiliations:** 1 Division of Pediatric Gastroenterology, Hepatology, and Nutrition, Department of Pediatrics, School of Medicine, The University of Jordan, Amman 11942, Jordan; 2 Jordan University Hospital, Amman 11942, Jordan; 3 School of Medicine, The University of Jordan, Amman 11942, Jordan; 4 Gastroenterology Interest Group (GIG), School of Medicine, The University of Jordan, Amman 11942, Jordan; 5 Department of Radiology and Nuclear Medicine, School of Medicine, The University of Jordan, Amman 11942, Jordan; 6 Department of Pediatrics and Neonatology, Faculty of Medicine, The Hashemite University, Zarqa 13133, Jordan; 7 Division of Gastroenterology and Hepatology, Department of Internal Medicine, School of Medicine, The University of Jordan, Amman 11942, Jordan

**Keywords:** gastrostomy tube, caregiver satisfaction, pediatric nutrition, home enteral nutrition, quality of life, SAGA-8 questionnaire, Jordan, percutaneous endoscopic gastrostomy PEG

## Abstract

**Background/Objectives:** Gastrostomy tube (GT) placement plays a vital role in managing children with chronic illnesses who are unable to meet their nutritional needs orally. While its clinical benefits are well established, limited data exist on caregivers’ satisfaction with GT use in Jordan. This study aimed to assess caregivers’ satisfaction and identify factors that influence their experiences by using a validated satisfaction scoring system in which a score greater than 20 indicates a high level of satisfaction. **Methods:** A cross-sectional study was conducted at Jordan University Hospital, including children under 18 years of age who underwent endoscopic GT insertion between July 2017 and December 2024. Caregivers completed the Structured Satisfaction Questionnaire with Gastrostomy Feeding (SAGA-8), and demographic and clinical data were collected. Statistical analyses explored associations between satisfaction levels and patient-, caregiver-, and healthcare-related factors. **Results:** A total of 46 caregivers participated. The median satisfaction score was 26.1, surpassing the high satisfaction threshold of 20. Overall, 63% of caregivers expressed satisfaction or high satisfaction with GT feeding, and 82.6% were satisfied with the support provided by the healthcare team. Additionally, 69.5% and 65.2% of caregivers reported improvements in their child’s nutritional status and overall family well-being, respectively. Notably, 89.1% observed a reduction in feeding time, and 84.8% reported fewer respiratory infections following GT placement. Over half of the caregivers (58.7%) indicated that they would have agreed to earlier GT placement if they had been more aware of its benefits. **Conclusions:** Caregivers reported high satisfaction with GT use, with scores well above the validated threshold indicating high satisfaction. These findings highlight the positive impact of GT placement on children’s health outcomes and family quality of life. Enhancing caregiver education and providing robust healthcare support are crucial to improving the management of children who require GT feeding.

## 1. Introduction

Home enteral nutrition (HEN) has become a cornerstone of care for pediatric patients with a functioning gastrointestinal tract who are unable to meet their nutritional needs orally [1]. This inability may arise from a variety of chronic or complex medical conditions, often leading to failure to thrive and necessitating specialized nutritional support [1,2,3]. HEN has been shown to improve nutritional status, decrease the frequency and duration of hospitalizations, enhance adherence to medical recommendations, and contribute to better overall health outcomes in this patient population [3,4,5]. Additionally, HEN allows families to provide life-sustaining nutritional support in the comfort of their homes, improving quality of life (QOL) for children and lowering the incidence of hospital-acquired complications, including infections [4,6].

Central to this process is the use of gastrostomy tubes (GT), which offer a reliable and long-term solution for enteral feeding. The European Society for Pediatric Gastroenterology, Hepatology, and Nutrition (ESPGHAN) recommends enteral feeding through gastrostomy, enterostomy, gastrojejunal, or jejunostomy tubes when oral intake remains insufficient for more than 4–6 weeks [7]. The endoscopic insertion of GTs has gained popularity due to its minimally invasive nature, shorter recovery times, and lower complication rates compared to traditional surgical methods [8,9]. This procedure has facilitated the transition to HEN, yet its success is closely tied to caregivers’ experiences and perceptions.

Gastrostomy tube placement not only impacts the QOL for pediatric patients but also significantly affects their caregivers. Pederson et al. demonstrated that caregivers of enterally fed children experienced notably higher stress levels [10]. This suggests that while GT placement alleviates some challenges, it does not fully relieve the burdens faced by children with GTs and their families [11].

The caregivers’ role in managing GTs and HEN is pivotal, as they are often responsible for the day-to-day oversight of feeding, ensuring adequate nutrition, and monitoring for complications [12]. Studies have shown that caregivers’ performance improves when they are more satisfied with the GT, as higher satisfaction rates are associated with improved nutritional status, a reduction in respiratory infections, and fewer hospitalizations [13]. Therefore, educating caregivers about GTs is essential, not only to address their concerns and increase satisfaction but also to minimize long-term complications, such as tube dislodgement, infection, and gastrointestinal issues, which require careful observation [14,15].

In the adult and pediatric populations, studies have shown that caregivers’ education about the use of GTs has improved their satisfaction with GT use [16,17,18,19]. Questionnaires were developed to objectively quantify and measure caregivers’ satisfaction with the GT placement process, including the Structured Satisfaction Questionnaire with Gastrostomy Feeding (SAGA-8), which was first developed in 2011 and has been widely used since then [20,21,22]. To the best of our knowledge, no previous studies from Jordan have objectively addressed caregivers’ satisfaction with GT use and the possible contributing factors to their attitudes toward using them.

This study aims to assess caregivers’ satisfaction with GT use in Jordanian children and to examine how it is influenced by factors related to the patients, their caregivers, and health care providers.

## 2. Materials and Methods

This cross-sectional study was conducted at JUH after obtaining approval from the Institutional Review Board committees of both JUH and the Jordan University School of Medicine. Using JUH electronic medical records, all children seen at the pediatric gastroenterology, hepatology, and nutrition clinic for PEG tube-related issues were identified. The study included all children aged less than 18 years who underwent endoscopic GT insertion at JUH between July 2017 and December 2024. Children were excluded from the study if their GTs were placed surgically or at centers outside JUH, if their parents could not be contacted by phone or declined participation, or if their data were incomplete.

Of the 63 eligible patients identified through electronic medical records, 13 caregivers could not be reached, despite repeated phone attempts over a 4-week period, three declined to participate, and one child was excluded due to GT placement outside our institution. These 17 cases were excluded from the final analysis. When available, basic demographic data (age and sex) were reviewed for excluded patients and showed no notable differences compared to the included participants. However, the potential for selection bias due to non-response is acknowledged. A total of 46 caregivers were eventually enrolled in the study and provided data on their children.

The study involved reviewing electronic medical charts to collect demographic and clinical data, including age; gender; GT size; date of insertion; duration of use; and the type of GT at the time of the study. GTs at JUH are of two types: the first step, or the percutaneous endoscopically placed GT (PEG), and the low-profile, skin-level, balloon-inflated GT. Caregivers of the children were contacted through phone surveys to assess their satisfaction with the GT. During the call, an information sheet was explained, and verbal consent was obtained before proceeding with the survey. The data collected during the calls were entered into a Google Form in real time. The calls were carried out by members of the medical team (D.S., Z.A.S., H.M.A., and N.O.) and supervised by the principal investigator (F.K.A.), none of whom had any prior connection to the patients or their caregivers.

The survey consisted of two main sections. The first section gathered demographic data about the parents, including their profession, education level, and monthly income. The second section utilized the Structured Satisfaction Questionnaire with Gastrostomy Feeding (SAGA-8) to evaluate caregiver satisfaction with GTs [20]. This questionnaire comprises eight questions: the first three assess caregivers’ perspectives regarding the acceptance of gastrostomy, the ease of its implementation, and the support provided by the hospital. The subsequent four questions focus on the patient, addressing the reduction in feeding time, the frequency of respiratory infections, the caregivers’ perceptions of changes in the child’s potential status and general health, and the family’s overall situation. The final question asks whether the caregiver would have agreed to gastrostomy insertion at an earlier stage if they had known its outcomes. Responses were measured using a 5-point Likert scale, ranging from 1 (“not satisfied at all”) to 5 (“very satisfied”), or a dichotomous scale, with scores of 2 for “Yes” and 1 for “No.” The total questionnaire score ranged from 8 to 31, with higher scores indicating greater satisfaction. A score greater than 20 was considered indicative of a high level of satisfaction [22].

The SAGA-8 questionnaire used in this study has been previously validated, demonstrating its reliability and appropriateness [20]. It was translated from English to Arabic to ensure accessibility for the target population. To maintain the accuracy and consistency of the content, a back-translation process was performed, in which the Arabic version was translated back into English to confirm that the original meaning was preserved.

Data were entered, recorded, and cleaned using Microsoft Excel. Statistical analyses were performed with IBM SPSS Statistics version 23. Descriptive statistics were used to summarize demographic and clinical characteristics, with categorical variables presented as frequencies and percentages and continuous variables presented as medians and interquartile ranges. The SAGA-8 questionnaire scores were analyzed to assess caregiver satisfaction. Comparative analyses were conducted to explore associations between categorical variables and satisfaction levels, using the Mann–Whitney U test for comparisons between two groups and the Kruskal–Wallis test for comparisons involving more than two groups. Spearman’s rank correlation was used to assess the association between continuous variables and satisfaction scores. A *p*-value of <0.05 was considered statistically significant.

## 3. Results

**Figure 1:**
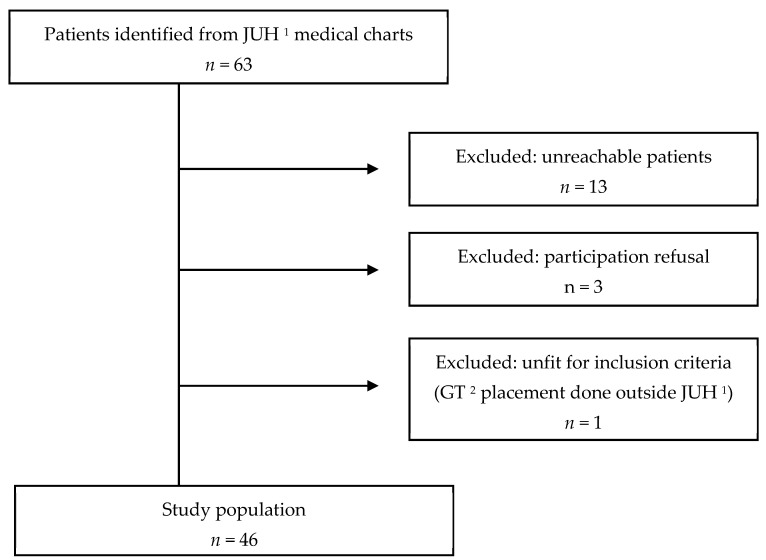
Flow diagram of caregiver enrollment. ^1^ JUH: Jordan University Hospital. ^2^ GT: gastrostomy tube.

Sixty-three patients were initially screened for inclusion. Thirteen caregivers could not be reached for study enrollment; three children were excluded from the study because their caregivers refused to participate in the study; and one child had his GT placed outside JUH. A total of 46 caregivers were eventually enrolled in the study and provided data on their children, as shown in Figure 1.

Among caregivers, 50% were fathers, 42.1% were mothers, 5.3% were uncles, and 2.6% were grandfathers. Male children accounted for 52.2% of the study population. The median age at the time of GT placement was 3 years (range: 3 months to 18 years), and the current median age was 7.7 years (range: 3.1 to 21.2 years).

The socioeconomic characteristics of patients’ families are summarized in Table 1.

The largest proportion of children (57.9%) had a low-profile skin-level GT at the time of the study (Table 1), with a median duration of use of 18 months (range: 0.25–84 months). Seven children had passed away from their primary neurological condition by the time this survey was conducted.

Table 2 presents caregivers’ responses to eight questions assessing their satisfaction with the GT from the SAGA-8 questionnaire.

The mean and median for the overall satisfaction score were (26.10). Most participants expressed positive experiences, with 63.0% reporting that they were satisfied or very satisfied with GT feeding and 76.1% reporting satisfaction with its management. Additionally, 82.6% were satisfied with the support provided by the center, while 69.5% and 65.2% reported satisfaction regarding improvements in their child’s nutritional status and overall family situation, respectively. A large majority of caregivers observed tangible benefits following GT placement: 89.1% noted a decrease in the time needed for feeding, and 84.8% reported fewer respiratory infections. A total of 58.7% of caregivers indicated that if they had been more aware of the benefits earlier, they would have consented to GT placement sooner. Caregivers of children who had passed away before this study was conducted reported high levels of satisfaction with GT during their children’s lives, with an overall satisfaction score of 23.3 (range: 19–29).

To evaluate the role of any contributing factors, caregiver satisfaction with GT was evaluated across a range of demographic and clinical factors (Table 3).

Satisfaction scores did not significantly differ between female and male children (*p* = 0.75) or between caregivers of children with PEG tubes (26.4 ± 7.75) and low-profile skin-level GTs (28 ± 4.75) (*p* = 0.31). Although fathers reported slightly higher satisfaction rates (28 ± 7) compared to mothers (27.5 ± 3.75), this difference did not reach statistical significance (*p* = 0.059). Other factors were not significant, as shown in Table 2. A correlation analysis revealed no significant relationship between caregiver satisfaction and age at GT placement (r = 0.027, *p* = 0.86). A moderate, non-significant positive correlation was observed between satisfaction and the duration of GT use (r = 0.297, *p* = 0.075), suggesting a potential trend for greater satisfaction with longer use. Overall, while a few trends were noted, demographic and caregiving factors showed limited influence on caregiver satisfaction, with all observed differences not reaching statistical significance (*p* > 0.05).

## 4. Discussion

This is the first study to address caregivers’ satisfaction with the use of GTs in Jordanian children, filling an important gap in the literature regarding this issue. A previous study demonstrated the safety and outcomes of GTs in Jordanian children, and this study helps to provide a broader perspective on caregivers’ experiences [23]. It is expected that this study will help healthcare providers better understand caregivers’ perspectives and the factors that affect their decision-making for the children under their care.

The findings in our study demonstrated a high level of caregiver satisfaction, with the majority reporting improvements in their children’s nutritional status, a reduction in feeding time, and fewer respiratory infections. These results align with international studies that have shown significant clinical benefits following GT placement [16,22,24]. This high satisfaction level can be attributed in part to the quality of care delivered at the treatment institution, where caregivers receive clear instructions, regular support, and close follow-up. The kindness and responsiveness of the staff, along with the low rate of complications observed after GT placement, likely made caregivers feel more comfortable and confident in caring for their children at home.

These clinical benefits are known to extend beyond direct health outcomes to positively influence family dynamics and caregiver well-being. This was evident in our study, with around two-thirds of caregivers reporting an improvement in the overall situation of their children and families following GT placement, which has also been confirmed in prior studies examining the psychosocial impact of GT placement [11,12]. These findings underscore the significant impact of GT placement on families and should encourage healthcare providers to dedicate more time to the care of children with GTs and educate their parents about GT management, as this represents time well spent.

Satisfaction rates among caregivers of children who had passed away prior to the study were also high in this study. This indicates that despite the eventual loss of their children due to the underlying disease, caregivers still viewed GT placement as a beneficial intervention that improved their children’s quality of life. These findings highlight the importance of offering GTs as a supportive measure in managing complex medical conditions. This is especially important in a country like Jordan, where cultural values emphasize family caregiving, emotional closeness, and a strong sense of duty to alleviate suffering. In this context, GT placement may be seen not only as a medical intervention but also as an act of care, love, and religiously guided compassion.

Although some authorities consider laparoscopic-assisted gastrostomy (LAG) insertion the safest method for GT placement in certain patient populations compared to the endoscopic approach, our study demonstrated a comparable caregiver satisfaction rate to that reported among caregivers whose children underwent LAG insertion, regardless of whether anti-reflux surgery was performed concurrently [25,26]. This can be attributed to the improvement in children’s overall condition following GT insertion and the high-quality follow-up care provided at the treatment center.

Because of these benefits, more than half of the caregivers in our study reported that they would have accepted GT placement earlier if they were made aware of its advantages. This finding is consistent with other studies that have emphasized the critical role of early and clear communication between healthcare providers and families to facilitate timely decision-making [13,16]. It also highlights the need for educational programs for both the caregivers and the healthcare providers to raise awareness about the long-term benefits of GT placement and the benefits gained from its presence.

A notable finding in this study was the high caregiver satisfaction rate (over 80%) with the support provided by the healthcare team. Previous studies have shown that comprehensive support and counseling significantly improve caregiver satisfaction and confidence in managing GT care [6,12,16]. This highlights the importance of ensuring healthcare provider availability for follow-up and addressing GT-related concerns. Establishing a dedicated “GT team”—where one member handles caregiver inquiries and triages them to a specialized GT nurse, who manages concerns and consults with the treating physician—can further enhance the caregiver experience [27,28]. Access to healthcare services—particularly specialized procedures like GT insertion—is not uniform across all regions in Jordan, making the positive experiences reported by caregivers at a tertiary care center even more significant. Their satisfaction reflects not just the clinical outcome but also the trust built through respectful communication, culturally sensitive counseling, and dependable follow-up.

In our study, caregiver satisfaction did not significantly differ based on demographic or socioeconomic factors such as income, parental education, or the child’s age and gender. However, Alsaggaf et al. suggested that socioeconomic factors may influence caregiver satisfaction [19]. Our findings suggest that satisfaction with GT placement is broadly perceived and consistent across different caregiver backgrounds, a finding that is supported by the validation studies of the SAGA-8 satisfaction tool [20,21].

Furthermore, satisfaction levels were comparable between caregivers of children with PEG tubes and those with low-profile skin-level GTs. This observation supports previous research indicating that both types of GT are effective and accepted by caregivers [14]. However, it remains important for healthcare providers to openly discuss the various gastrostomy options available at their institutions, as caregiver preferences can vary. For instance, the push technique used to insert low-profile, skin-level GTs was not available at our center until recently. A few caregivers specifically requested this method once it became available. Ongoing follow-up will be valuable to assess their satisfaction with this technique over time.

Although the correlation between the duration of GT use and satisfaction was not statistically significant, a moderate positive trend was observed, suggesting that caregivers become more confident and satisfied as they adapt to managing GT over time. Similar patterns have been reported in the literature, with a recent meta-analysis by Prakash et al. showing that prolonged exposure to GT care may reduce anxiety and improve overall satisfaction [29].

Future research should explore the long-term quality of life outcomes for both children with GTs and their caregivers, including the psychological, social, and economic impacts of home enteral nutrition. Previous studies have emphasized the importance of integrating quality of life assessments into care models for patients and families managing chronic conditions that require GTs [11]. Additionally, the development of structured national programs to support caregivers may further enhance satisfaction and clinical outcomes.

While this study provides valuable insights into caregivers’ experiences with pediatric gastrostomy tubes, certain limitations should be noted. As a single-center study with a relatively small sample size, the findings may not fully represent the experiences of all caregivers in different settings. Additionally, relying on self-reported data may introduce some degree of recall or response bias. However, using a validated questionnaire may have helped to reduce the impact of such bias. Although every effort was made to reach all eligible participants, the exclusion of caregivers who were unreachable or declined participation might have influenced the diversity of the captured perspectives. Although the use of a validated questionnaire and anonymized data collection helped to reduce bias, the possibility of social desirability in caregiver responses cannot be entirely ruled out. To address this issue, interviews were conducted by non-clinical team members, and caregivers were assured that their participation would not affect their child’s care. Furthermore, conducting the interviews by telephone may have increased the likelihood of social desirability bias, as caregivers might have felt pressure to respond favorably during direct verbal interaction.

Despite these limitations, the study offers important preliminary data that can help guide future research and improve caregiver support programs. Larger, multi-center studies incorporating more diverse caregiver populations and objective outcome measures are encouraged to further validate and expand upon these findings. Hospitals implementing GT care pathways should ensure early caregiver engagement, preferably before nutritional decline becomes severe. Institutions should also consider offering flexible GT insertion methods to accommodate caregiver preferences.

## 5. Conclusions

This study highlights a high level of caregiver satisfaction with GT placement among Jordanian children, particularly in terms of improved nutrition, reduced feeding burden, and enhanced quality of life. Even among caregivers of children who later passed away, GT placement was perceived as beneficial. These findings underscore the importance of early, clear communication and structured follow-up to support families throughout the GT care journey. Cultural and societal factors, including strong familial roles and religious values, likely influence caregiver perceptions and should be considered in counseling and care planning. The results emphasize the need for healthcare systems to ensure equitable access to GT services, provide comprehensive caregiver education, and develop support mechanisms that address psychological and social needs. Future multicenter studies are encouraged to further explore long-term outcomes and refine care pathways for pediatric patients who require enteral feeding.

## Figures and Tables

**Table 1:** Patients’ baseline characteristics and caregivers’ socioeconomic profiles.

Variable	Category	*n* (%)
Age at GT placement (years)	Median ± IQR	3 ± 6.63
Child’s sex	Male	24 (52.2)
	Female	22 (47.8)
GT type	Low-profile skin-level GT	24 (57.9)
	PEG tube	22 (42.1)
Family monthly income (JD)	<500	20 (43.5)
	500–1500	24 (52.2)
	>1500	2 (4.3)
Father’s occupational status	Private sector	21 (45.7)
	Public sector	19 (41.3)
	Unemployed	2 (4.3)
	Non-supporting parent	1 (2.2)
	Deceased	1 (2.2)
	Other	2 (4.3)
Father’s educational level	No formal education	1 (2.2)
	Primary school	3 (6.5)
	Middle school	4 (8.7)
	High school	16 (34.8)
	Bachelor’s degree	22 (47.8)
Mother’s occupational status	Housewife	36 (78.3)
	Public sector	6 (13.0)
	Private sector	4 (8.7)
Mother’s educational level	No formal education	1 (2.2)
	Primary school	3 (6.5)
	Middle school	4 (8.7)
	High school	21 (45.6)
	Bachelor’s degree	17 (37.0)

GT: gastrostomy tube; IQR: interquartile range; PEG: percutaneous endoscopic gastrostomy; JD: Jordanian dinar.

**Table 2:** Caregivers’ satisfaction with the gastrostomy tube according to the SAGA-8 questionnaire.

**Section A: Likert-Scale Questions (Items 1–5)**						
Question number	Question	1 (*n*, %)	2 (*n*, %)	3 (*n*, %)	4 (*n*, %)	5 (*n*, %)
1	How do you rate your satisfaction with GT feeding?	4 (8.7)	1 (2.2)	12 (26.1)	4 (8.7)	25 (54.3)
2	How do you evaluate GT management?	0 (0.0)	2 (4.3)	9 (19.6)	9 (19.6)	26 (56.5)
3	How do you evaluate the support offered by our center?	1 (2.2)	1 (2.2)	6 (13.0)	8 (17.4)	30 (65.2)
4	How do you perceive your child’s change in nutritional status?	3 (6.5)	2 (4.3)	9 (19.6)	10 (21.7)	22 (47.8)
5	How do you rate the change in your child and your family’s status?	2 (4.3)	2 (4.3)	12 (26.1)	11 (23.9)	19 (41.3)
**Section B: Binary Yes/No Questions (Items 6–8)**						
Question number	Question	Yes (*n*, %)			No (*n*, %)	
6	Has the time necessary for feeding decreased?	41 (89.1)			5 (10.9)	
7	Has the number of respiratory infections decreased?	39 (84.8)			7 (15.2)	
8	Would you have accepted earlier GT placement, knowing its benefits?	27 (58.7)			19 (41.3)	

SAGA-8: Structured Satisfaction Questionnaire with Gastrostomy Feeding; GT: gastrostomy tube. Likert scale meaning: 1 = Not satisfied at all; 2 = Somewhat satisfied; 3 = Neutral; 4 = Satisfied; 5 = Very satisfied. Binary scale: Yes = 2; No = 1.

**Table 3:** Demographic and clinical factors evaluated for association with caregiver satisfaction with the gastrostomy tube.

Variable	Category	Median Satisfaction Score (± IQR)	*p*-Value
Child’s sex	Female	26.5 ± 6.75	0.75
	Male	27.0 ± 6.25	
GT type	PEG tube	26.4 ± 7.75	0.25
	Low-profile skin-level GT	28.0 ± 4.75	
Contacted caregiver	Father	28.0 ± 7.0	0.059
	Mother	27.5 ± 3.75	
	Other (uncle/grandfather)	21.0 ± 5.5	
Mother’s occupational status	Housewife	26.0 ± 8.0	0.20
	Employed (public/private)	28.0 ± 2.25	
Father’s occupational status	Public sector	28.0 ± 5.0	0.067
	Private sector	26.0 ± 5.5	
	Other (e.g., deceased/unemployed)	21.5 ± 10.75	
Parental education	≥1 parent with higher education	27.0 ± 5.0	0.94
	Neither parent with higher education	27.0 ± 8.5	
Monthly family income (JDe)	<500	26.0 ± 7.75	0.40
	≥500	27.0 ± 6.0	
	Correlation Coefficient		
Age at GT placement (years)	r = 0.027		0.86
Duration of GT use (months)	r = 0.297		0.075

IQR: interquartile range; *p*-values were calculated using the Mann–Whitney U test for two-group comparisons, the Kruskal–Wallis test for >2 groups, and Spearman’s rank correlation for continuous variables; GT: gastrostomy tube; PEG: percutaneous endoscopic gastrostomy; JD: Jordanian dinar.

## Data Availability

The original contributions presented in this study are included in the article. Further inquiries can be directed to the corresponding author.

## Abbreviations

The following abbreviations are used in this manuscript:

GT	Gastrostomy Tube
PEG	Percutaneous Endoscopic Gastrostomy
HEN	Home Enteral Nutrition
QOL	Quality of Life
ESPGHAN	European Society for Pediatric Gastroenterology, Hepatology, and Nutrition
JUH	Jordan University Hospital
SAGA-8	Structured Satisfaction Questionnaire with Gastrostomy Feeding
IQR	Interquartile Range
JD	Jordanian Dinar
SPSS	Statistical Package for the Social Sciences
